# Molecular breeding of thermo-sensitive genic male sterile (TGMS) lines of rice for blast resistance using *Pi2* gene

**DOI:** 10.1186/s12284-015-0048-3

**Published:** 2015-02-12

**Authors:** Jiefeng Jiang, Tongmin Mou, Huihui Yu, Fasong Zhou

**Affiliations:** National Key Laboratory of Crop Genetic Improvement, National Center of Plant Gene Research (Wuhan), Huazhong Agricultural University, Wuhan, 430070 China; Life Science and Technology Center, China National Seed Group Co., Ltd., Wuhan, 430206 China

**Keywords:** Rice blast resistance, TGMS line, Two-line hybrid rice, Marker-assisted selection, *Pi2*

## Abstract

**Background:**

Blast disease caused by the fungal pathogen *Magnaporthe oryzae* is one of the big problems in rice production in China, especially for high yield hybrid varieties made from a two-line system in which thermo-sensitive genic male sterile (TGMS) lines are used. In this study, we report the introgression of a rice blast resistance gene *Pi2* from VE6219 into C815S, an elite rice TGMS line, leading to the development of blast resistant TGMS lines through marker assisted selection (MAS) and phenotypic selection approaches.

**Results:**

Four new TGMS lines with blast resistance gene *Pi2* were developed from C815S (an elite TGMS line susceptible to the blast, used as recurrent parent) and VE6219 (a blast resistant line harboring *Pi2*, used as donor parent). The pathogenicity assays inoculated with 53 blast prevalent isolates in glasshouse showed that the blast resistant frequency of the four TGMS lines was 94.3%-98.1% that is equivalent to blast resistant donor parent VE6219. The field evaluation of the new lines and hybrids made from them at a blast epidemic site also showed high resistant levels against the blast. The genetic background of the newly developed TGMS lines were examined using a whole-genome single nucleotide polymorphism (SNP) array (RICE6K) that turned out more than 83% of the genomic markers were derived from the recurrent parent. The critical temperature points of fertility-sterility alteration of the new TGMS lines were between 22°C and 23°C of daily mean temperature, which is similar to that of C815S. The complete male sterility under natural growth conditions at Wuhan last more than 80 days. Their agronomic and grain quality traits meet the requirement for two-line hybrid rice production.

**Conclusions:**

The broad-spectrum and durable rice blast resistant gene *Pi2* was introgressed into the elite TGMS line C815S background. The newly developed TGMS lines can be practically used for two-line hybrid rice breeding and must play an important role in sustainable rice production in China.

## Background

Rice blast caused by *Magnaporthe oryzae* is the most destructive diseases widely prevalent in rice fields, leading to significant grain yield and quality reduction. The disease including leaf and panicle blast can cause yield losses from 10% to 30% in severely infected crops (Skamnioti and Gurr 2009). It has been a major problem for hybrid rice production due to the large area monoculture of a crop variety and quickly evolved major race of the pathogene (Bonman et al. 1986; Liu et al. 2010).

So far, 86 blast resistance genes have been documented (Liu et al. 2013). A few of them have already been incorporated into rice varieties, which are now widely cultivated in many countries. The rice blast resistance gene *Pi2* was introduced from a resistant *indica* rice cultivar 5173 into a susceptible cultivar CO39, and the derived isogenic line was named C101A51 (Mackill and Bonman 1992). At least six resistance genes (*Pi2, Pi9, Piz-t, Piz, Pigm* and *Pi50*) were mapped at the same location, close to the centromere of chromosome 6 (Deng et al. 2006; Hayashi et al. 2004; Liu et al. 2002; Zhu et al. 2012). *Pi2* is one of the broad spectrum resistance genes highly effective in rice blast control. Lines carrying *Pi2* were tested with 43 blast isolates collected from 13 countries and showed resistant to 36 of them (Liu et al. 2002). *Pi2* gene has been isolated and characterized, which encodes a NBS-LRR (a nucleotide-binding site and leucine-rich repeat) protein (Zhou et al. 2006). Marker-assisted selection (MAS) has become an important approach to the development of disease resistance cultivars, especially in the utilization of cloned genes, which largely reduces time and cost in breeding programs (Ishihara et al. 2014; Jiang et al. 2012; Narayanan et al. 2002). In this study we employed MAS to develop thermo-sensitive genic male sterile (TGMS) lines by introducing *Pi2* into the TGMS background.

Comparing with three-line rice hybrid production system, two-line system with TGMS has some advantages: (1) The male sterility were controlled by recessive nuclear genes. Any genotype with good combining ability can be used as a male parent. The frequency of obtaining heterotic hybrids in testcrosses is relatively higher than three-line system. (2) It can be used to develop hybrids of *japonica* or basmati in which there are very low frequency of cytoplasmic male sterile (CMS) restorers. (3) It is especially suited for developing sub specific hybrid (*indica*/*japonica*) because there is no restriction of restoring-maintaining relationship in TGMS system. (4) The cost for hybrid seed production is lower as compared to the three-line system. Two-line hybrid breeding has been widely used in China as an effective approach to the improvement of rice grain yield and quality. However, there is an urgent need to enhance its blast disease resistant. Here we report the line breeding process and main results in the development of TGMS lines with broad spectrum resistance to blast through MAS, and the subsequent check of their genetic background with the aid of a rice genomic SNP array.

## Results

### Introgression of rice blast resistance gene *Pi2* by MAS

**Figure 1:**
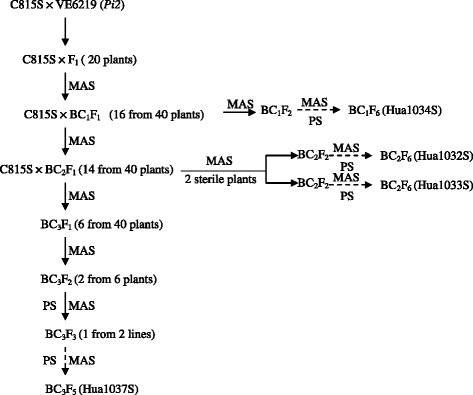
**The scheme showing the development of rice blast resistant TGMS lines by introgression of rice blast resistant genes** ***Pi2*** **(donor parent: VE6219) into the susceptible popular TGMS recipient parent, C815S, through MAS approaches.** MAS: marker assistance selection; PS: phenotypic selection.

**Figure 2:**
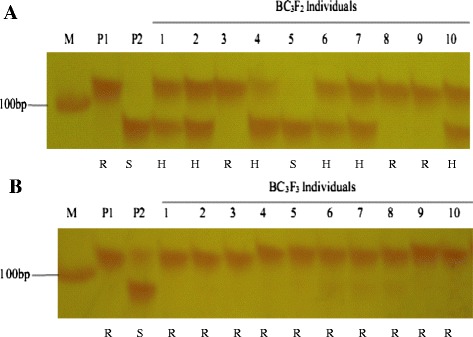
**Marker co-dominant and polymorphic for** ***Pi2*** **in the populations. (A)** marker RM527 for *Pi2* were fractionated on 4% polyacrylamide gel in the BC_3_F_2_ population, and **(B)** in the BC_3_F_3_ population. M: 50 bp DNA Marker; P_1_: VE6219 (*Pi2* donor parent); P_2_: C815S; lanes 1–10: 10 Plants of the BC_3_F_2_ or BC_3_F_3_ populations. R, S and H stand for the homozygous resistant, homozygous susceptible and heterozygote genotypes, respectively.

F_1_ plants obtained from the cross of C815S (an elite TGMS line) and VE6219 (carrying *Pi2*), were screened for presence of the target resistance gene *Pi2* using the gene linked molecular marker RM527 to identify the true F_1_s showing heterozygous and were backcross with C815S in summer season of 2008 at Wuhan. Of 40 BC_1_F_1_ plants, 16 individual plants were identified to be positive for *Pi2* using the marker RM527. In BC_1_F_1_, two selection strategies were taken in the subsequent generations (Figure 1). One is to select positive fertile plants and to be used for further backcrossing with C815S. Other is to select male sterile plants that would be ratooned under low temperature for harvesting BC_1_F_2_ seeds. The advanced backcross progenies of BC_2_ and BC_3_ were generated from the crosses of selected positive BC_1_F_1_ (16 plants from 40) and BC_2_F_1_ (14 plants from 40) by MAS (Figure 1). The same method was repeated for advancing the next generation to reach BC_2_F_2_ and BC_3_F_2_. The male sterile plants with homozygous *Pi2* were selected in BC_1_F_2_, BC_2_F_2_ and BC_3_F_2_ populations and ratooned under low temperature to obtain next generation seeds. Finally, we developed four TGMS lines that harbored the homozygous marker linked to *Pi2* and also showed desirable agronomic traits in different backcross generations, which were designated as Hua1034S (BC_1_F_6_), Hua1032S (BC_2_F_6_), Hua1033S (BC_2_F_6_), and Hua1037S (BC_3_F_6_) (Figure 1 and Figure 2).

### Genetic background examination of the newly developed TGMS lines

**Figure 3:**
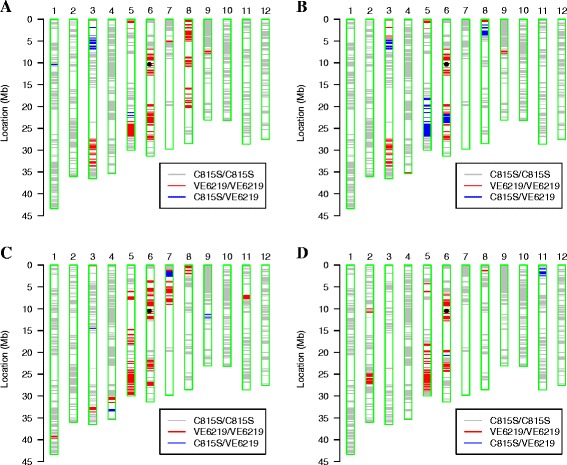
**Haplotype maps of genetic background profiling using RICE6K array. (A)** Hua1032S, **(B)** Hua1033S, **(C)** Hua1034S and **(D)** Hua1037S. The dots indicate the position of *Pi2* on chromosome 6.

In this study, RICE6K, a whole-genome single nucleotide polymorphism (SNP) array was used to to analyze the genetic background of the newly developed TGMS lines. The identified high quality markers, 1256 out of 5102, showed polymorphisms between the recurrent parent, C815S, and the donor parent, VE6219. The genetic background recovery of the recurrent parent in Hua1032S, Hua1033S, Hua1034S and Hua1037S were 83.93%, 88.47%, 83.15% and 88.93%, respectively, measured by percentage of the polymorphic marker ratios (Figure 3). All four TGMS lines had large fragments of chromosome 6 because of foreground *Pi2* selection in each generation by MAS. These four TGMS lines must be further self-pollinated and selected since several fragments in each TGMS line were still heterozygous.

### Evaluation of blast resistance

Leaf blast resistances of the newly developed TGMS lines and the two parents were assessed using 53 isolates of *M. oryzae* from Zhejiang and Guangdong provinces, China, in greenhouse. The donor parent VE6219, containing *Pi2* gene, showed broad-spectrum resistance to rice blast with a high resistance frequency of 90.6%, and the recurrent parent C815S was found susceptible to the most of isolates with the resistance frequency of 34.0%. The developed TGMS line Hua1037S was resistant against 50 of 53 blast isolates with a resistance frequency of 94.3% slight higher resistance frequency as of VE6219. Other improved TGMS lines viz. Hua1032S, Hua1033S and Hua1034S with 98.1% resistance frequencies showed higher resistance frequency than VE6219 (Table 1).

**Table 1:** **Disease resistance reaction of newly developed TGMS lines (NT), recipient (RP) and donor parents (DP) along with susceptible check (SC) variety to 53 rice blast isolates under artificial inoculation in greenhouse**

**Blast isolates**	**C815S**	**Hua1032S**	**Hua1033S**	**Hua1034S**	**Hua1037S**	**VE6219**	**CO39**
A13	R	R	R	R	R	R	S
A15	R	R	S	R	R	S	S
B01	S	R	R	R	R	R	S
B05	S	R	R	R	R	R	S
B09	S	R	R	R	R	R	S
B13(1)	R	R	R	R	R	R	S
B13(2)	S	R	R	R	R	R	S
B13(3)	S	R	R	R	R	R	S
B13(4)	S	R	R	R	S	R	S
B13(5)	S	R	R	R	R	R	S
B13(6)	S	R	R	R	R	R	S
B13(7)	S	R	R	R	R	R	S
B13(8)	S	R	R	R	R	R	S
B13(9)	S	R	R	R	R	R	S
B13(10)	R	R	R	R	R	S	S
B13(11)	S	R	R	R	R	R	S
B15(1)	S	R	R	R	R	R	S
B15(2)	S	R	R	R	R	R	S
B15(3)	R	R	R	R	R	R	R
B15(4)	R	R	R	R	R	R	R
B15(6)	R	R	R	R	R	R	R
B15(7)	S	R	R	R	R	R	S
C13(1)	S	R	R	R	R	R	S
C13(2)	S	R	R	R	R	R	S
C13(3)	R	R	R	R	R	R	S
C13(4)	S	R	R	R	R	R	S
C13(5)	R	R	R	R	R	R	S
C13(6)	R	R	R	R	R	S	S
C13(7)	R	R	R	S	S	S	S
C15(1)	S	R	R	R	R	R	S
C15(2)	S	R	R	R	R	R	S
C15(3)	R	R	R	R	R	R	S
G01	R	R	R	R	R	R	S
B9(11–109)	S	R	R	R	R	R	S
C15(12–103)	R	R	R	R	R	R	S
C15(H-1)	S	R	R	R	R	R	S
C15(10–151)	S	S	R	R	R	R	S
B9(09-78-20)	S	R	R	R	R	R	S
C15(12–217)	S	R	R	R	R	R	S
C15(12–211)	S	R	R	R	R	R	S
G1(10–85)	S	R	R	R	R	R	S
C9(06–113)	R	R	R	R	S	R	S
C9(12–180)	S	R	R	R	R	R	S
B9(06–234)	S	R	R	R	R	S	S
B17(12–105)	S	R	R	R	R	R	S
D5(12–13)	S	R	R	R	R	R	S
G1(09–135)	S	R	R	R	R	R	S
C3(11–216)	R	R	R	R	R	R	S
C3(H-3)	R	R	R	R	R	R	S
D5(H-2)	S	R	R	R	R	R	S
C9(06–263)	S	R	R	R	R	R	S
E3(11–109)	R	R	R	R	R	R	S
B9(10–101)	S	R	R	R	R	R	S
NO. of resistant isolates	18	52	52	52	50	48	3
NO. of susceptible isolates	35	1	1	1	3	5	50
Resistance frequency (%)	34.0	98.1	98.1	98.1	94.3	90.6	5.7

In order to evaluate the blast resistance of four TGMS lines and their derived hybrids in field, plants of each line were grown at Wangjia village of Yuan-An county, Hubei, China, where the altitude is about 600 meters, a hilly region with frequent blast epidemic in rice growing season, and it is a test site area for evaluation of the blast resistance of the China national rice regional test varieties. The results (Table 2) showed that C815S was susceptible to rice blast with 7 score of leaf blast and 100% of neck blast incidence, respectively. The leaf blast scores and neck blast incidences of the four TGMS lines were less than 1 and 6%, respectively, similar to donor parent VE6219. Two pollen parents (IR24 and R1005) and C815S’ derived hybrids were susceptible to both leaf and neck blast. However, the hybrids derived from the four newly developed TGMS lines were resistant against leaf and neck blast (Table 2).

**Table 2:** **The performance of leaf and neck blast resistances for the four newly developed TGMS lines, their derived hybrids and control materials under the natural field of blast epidemic area**

**Entries**	**Score of leaf blast**	**Incidence of neck blast (%)**
Hua1032S	0	6.0
Hua1033S	0	5.0
Hua1034S	1	0.0
Hua1037S	0	3.0
C815S	7	100.0
CO39	9	100.0
VE6219	0	5.0
Hua1032S/IR24	3	11.0
Hua1033S/IR24	0	11.0
Hua1034S/IR24	0	6.0
Hua1037S/IR24	4	10.0
C815S/IR24	5	29.0
IR24	7	99.0
Hua1037S/R1005	0	0.0
Hua1032S/R1005	0	8.0
Hua1033S/R1005	0	6.0
Hua1034S/R1005	0	0.0
C815S/R1005	4	39.0
R1005	4	54.0

### Characterization of fertility-sterility alteration in growth chamber

The critical temperature point (CTP) of fertility-sterility alteration has practical importance especially for a TGMS line to safe seed production. In this study, the plants were placed in five growth chambers while the daily mean temperatures (DMT) were set for 21°C, 22°C, 23°C, 24°C and 25°C from 5–16 days after panicle initiation primordial stage for 12 days. The pollen grains collected from top five spikelets from each panicle per plant that headed during 5–16 days after the end of controlled growth chamber environment treatment were observed under microscope. The results showed that the pollen of the recurrent TGMS line i.e. C815S, was completely sterile (pollen sterility more than 99.5%) in the growth chambers with 22°C onwards to 25°C of DMT, but became partial fertile under 21°C, while the new TGMS lines, Hua1037S and Hua1034S had similar CTP of fertility-sterility alteration located in between 21°C and 22°C of DMT (Table 3). Therefore, it will be safe to carry out two-line hybrid seed production under conditions where DMT is higher than 22°C. However, the pollen sterility of Hua1032S and Hua1033S were less than 99.5% under 23°C while completely sterile in growth chambers with 24°C to 25°C DMT (Table 3). So, the natural DMT should be higher than 24°C for seed production safely.

**Table 3:** **Fertility-sterility alteration behavior of the newly developed TGMS lines and the recurrent parent under five different temperature regimes in growth chambers**

**Entries**	**Pollen sterility (%)**				
	**21°C**	**22°C**	**23°C**	**24°C**	**25°C**
Hua1032S	84.39	96.2	97.1	100.0	100.0
Hua1033S	81.29	95.3	99.4	100.0	100.0
Hua1034S	91.27	99.7	100.0	100.0	100.0
Hua1037S	86.48	100.0	100.0	100.0	100.0
C815S	69.62	99.5	100.0	100.0	100.0

**Figure 4:**
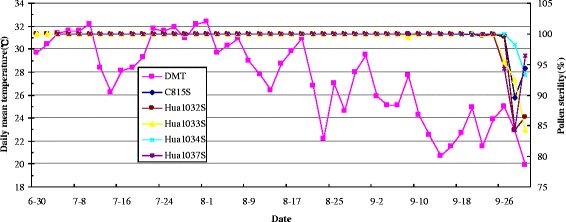
**The dynamic pollen fertility expressions of the newly developed TGMS lines and C815S relative to daily mean temperature (DMT) data from 8th July to 1st October in 2012 at Wuhan.**

In China, a practical usable TGMS line needs a stable sterility period of longer than 30 days in given region. We sowed TGMS lines at 15-day intervals at the Experimental Farm of Huazhong Agricultural University (HAU). The pollen fertilities in each line from the top five spikelets of primary panicles were investigated dynamically with two-day intervals under microscope from 30 June to 1 October. The pollen sterility data were recorded with average of five panicles from each line. The results showed that the pollen sterile periods of Hua1032S and Hua1033S were 88 days from 30 June to 26 September. Those of Hua1034S and Hua1037S were 80 days from 8 July to 26 September (Figure 4). Analysis of pollen sterility data in relation to temperature weather charts showed that when the DMT during 13 to 16 of September declined to below 22°C, the pollen grains of developed TGMS lines and C815S had shown reversal to partial fertility around 26 September. These phenomena indicated that the sensitive stage to temperature located at 13 days before heading. Although the DMT declined to 22.2°C on 23 August, the pollen of TGMS lines remained completely sterile, indicating their CTPs under natural conditions being similar as in growth chamber (Table 3 and Figure 4).

### Evaluation of agronomic and rice quality traits of the newly developed TGMS

Four newly developed TGMS lines were evaluated for their agronomic and grain quality traits as compared to C815S at the Rice Breeding Station of Hainan in the winter season 2011–2012. The results (Table 4) showed non-significant differences for the days from sowing to heading, number of panicles per plant, 1000-grain weight, milling rice percentage, grain length and grain length-width ratio between the newly developed TGMS lines and C815S. The plant height of Hua1034S was higher than C815S by 6.5 cm. The panicle lengths of all four newly developed TGMS lines were longer than that of C815S by 2.1-3.7 cm. The number of grains per panicle of Hua1033S and Hua1034S were more than that of C815S. The spikelet fertility of Hua1032S and Hua1033S, the very important trait in TGMS line multiplication, were 22-31% more than that of C815S. The gel consistency of all the developed TGMS lines were increased 15–29 mm compared with the recurrent parent, C815S. Another desirable grain quality trait was higher amylose content, thus increased taste for customer in China.

**Table 4:** **The performance of main agronomic and grain quality traits of newly developed blast resistant TGMS lines and C815S**

**TGMS line**	**DTH** ^**a**^	**PH (cm)**	**PN**	**PL (cm)**	**NGP**	**SF (%)**	**GW (g)**	**MRP (%)**	**RGL (mm)**	**L/W**	**GC (mm)**	**AC (%)**
Hua1032S	93.0	76.1	13.2	24.4**	145.8	63.6**	25.0	65.4	6.3	2.8	63.5**	12.7*
Hua1033S	93.0	75.9	12.6	23.5**	167.1*	72.6**	25.2	66.2	6.3	2.8	67.5**	12.2*
Hua1034S	97.0	83.7*	13.0	24.5**	160.9*	38.0	25.6	68.2	6.4	2.8	62.0**	15.0*
Hua1037S	96.0	72.6	12.8	23.1*	150.9	47.1	25.9	66.9	6.5	2.7	76.5**	15.4*
C815S	96.0	77.2	11.0	21.0	137.2	41.5	23.7	65.7	6.4	2.7	47.5	10.4

^a^DTH: days to heading, PH: plant height (cm), PN: panicle number, PL: panicle length (cm), NGP: number of grains per panicle, SF: spikelet fertility (%), GW: 1,000-grain weight (g), MRP: milling rice percentage, RGL: rice grain length, L/W: grain length-width ratio, GC: gel consistency, AC: amylose content, *, **Symbols followed after means indicate significant at the 5% or 1% significance level by the *T*-test.

## Discussion

The two-line system for hybrid rice production has been widely used in China for increasing rice grain yield. However, the prevalence of rice blast disease is jeopardizing the expansion of the popular hybrids as a result of their susceptibility to the disease. Thus, to enhance the blast resistance of the parental lines is essential for sustainable utilization of the high-yielding hybrids. In this study, we sought to improve rice blast resistance in C815S because several commercial hybrids using C815S as the female parent were widely grown in the *indica* rice growing area of the Central and Southern China. The closely linked and diagnostic marker RM527 was used for tracking *Pi2* in our breeding program, and the resistance gene was incorporated into C815S using backcrossing strategy. The newly developed TGMS lines, Hua1032S, Hua1033S, Hua1034S and Hua1037S, and the hybrids produced thereby, exhibited an elevated blast resistant. Our study clearly indicated that *Pi2* gene has a broad spectrum resistance to rice blast and is valuable for development of blast resistance rice hybrids. MAS-based backcross schemes were shown effective in the improvement of TGMS line for the blast resistance.

The genetic background of the four selected TGMS lines was examined by using a genomic SNP array, RICE6K. The results showed that the genetic background was not recovered to the recurrent parent levels although 3 cycles of backcrosses were performed. To recover a maximum recurrent parent genetic background in a short time, breeders would need a large backcross population and do background selection on top of the foreground check for the target gene (Rajpurohit et al. 2011). Suh et al. (2013) reported more than 92.1% recovery of background of recurrent parent Mangeumbyeo in BC_3_F_5_ with phenotypic selection without marker assisted background selection (MABS) during introgression of three bacterial blight resistance genes. In this project, we tried to develop new TGMS lines for stronger resistance against blast and with better agronomic traits to increase yields of the hybrid seeds, and our selections on the target traits obviously resulted in genetic drags. In our ongoing breeding program, we are using RICE6K to examine the genetic background of the selected lines and to more precisely remove the negative genetic drags. Interestingly, Hua1034S derived from BC_1_F_1_ contains 83.15% of genetic loci from C815S, similar to that of Hua1032S (83.93%) and Hua1033S (88.47%), which were derived from BC_2_F_1_ plants, and Hua1037S (88.93%), which was derived from a BC_3_F_1_ plant (Figure 1). It might be interpreted as that the recurrent genetic background was recovered faster than theoretically expected in early backcross generations because of selection on morphologic traits of the recurrent parent. In contrary, the recurrent genetic background was recovered slower than theoretically expected in higher backcross generations because of genetic drag of the introgressed gene, *i.e. Pi2*. With the aid of a whole-genome SNP array, the negative genetic drag would be removed more efficiently and the genetic background of a recurrent parent would be recovered quicker in higher backcross generations. The desirable allele *tms* loci of C815S was difficult to track using the linked molecular markers. Up to now, the *tms* gene/s in C815S has not been identified yet although several other *tms* genes in TGMS rice have been mapped (Hussain et al. 2012), and the *tms* gene mapping results were influenced by background, even they had same *tms* gene source, e.g. Guangzhan63S and Nongken58S (Ding et al. 2012; Liu et al. 2001; Lu et al. 2005; Xu et al. 2011; Zhang et al. 1994).

Heading date, a critical trait that determines cropping seasons and regional adaptability in rice, depends strongly on photoperiodic responses. More than 16 genes/QTLs have been identified as being involved in the photoperiodic flowering pathway in rice (Matsubara et al. 2008; Yano et al. 2001). *Hd1*, a major photoperiod sensitivity gene on chromosome 6, was tightly linked to a blast resistance gene, *Pi-z*^*t*^ (Yano et al. 1997; Yano et al. 2000), the allele at *Pi2*/*Pi9* (Zhou et al. 2006). In our experiments, many combinations from C815S used as female parent were sensitive to photoperiod because they did not heading in normal season growth duration (data not shown). However, the combinations from the above same pollen parents and the newly developed TGMS lines harboring *Pi2* gene could head like other normal varieties. These phenomena implied that no photoperiod sensitive gene replaced the photoperiod sensitive gene in C815S simultaneously when we introgressed the chromosome fragment including *Pi2* gene from nonphotoperiod sensitive variety VE6219 into C815S.

Conventional disease resistance breeding is hard sledding, time-consuming, blindness compared with MAS which offers a powerful strategy to transfer genes from exotic germplasm into cultivated lines. It is important to note that introgression genes in rice should not lead to yield penalty or other drawbacks on important agronomic traits. But MAS only focused on one or several genes, several traits, ignore the comprehensive traits, so we should transvalue the agronomic traits of selected lines. The recently designed rice genomic SNP arrays (Yu et al. 2014; Chen et al. 2014) have offered molecular breeders excellent tools for the genetic background selection which could significantly reduce the recurrent cycles of backcrossing and help to break the negative genetic linkage between a target gene and poor agronomy trait. For TGMS lines, the most important trait is CTP of fertility-sterility alteration which related to safe hybrid rice seed and self seed production. In China, a TGMS line could not be released if its CTP was higher than 24°C of DMT. Seed multiplication will be very difficult if the CTP of a TGMS line was below 21°C of DMT. In our study, the CTPs of Hua1034S and Hua1037S were located at 22°C of DMT similar to the recurrent parent, C815S. However, Hua1032S and Hua1033S were located at 23°C of DMT. So, Hua1034S and Hua1037S can be used for hybrid seed production when the DMT during booting stage is over 22°C and can be used for TGMS line seed multiplication when below 22°C. However, Hua1032S and Hua1033S can be used for hybrid seed production when the DMT during booting stage is over 23°C and can be used for seed multiplication when below 23°C. Finally, Hua1037S was considered as excellent female parent for two-line blast resistant hybrid breeding because of its blast resistance, low CTP and better agronomic traits.

## Conclusions

Rice blast disease is one of the most serious worldwide problems for rice production. Recently, it has become more serious and outspread, which urgently requires the development of rice blast resistant varieties. We successfully introgressed a broad-spectrum and durable rice blast resistance gene *Pi2* into an elite TGMS line to develop four blast resistance TGMS lines by using a dual-selection strategy of phenotypic and genotypic selection along with background screening to isolate improved breeding lines. These developed TGMS lines will be practical valuable for developing blast resistance two-line system hybrid rice in China.

## Methods

### Plant materials

C815S, an elite TGMS line widely used as female parent in two-line system hybrid rice breeding in China, but susceptible to blast was used as TGMS gene donor and recurrent parent. VE6219, a blast resistance breeding line (from the offspring between T1007 and C101A51) possessing *Pi2* gene, was used for blast resistance gene donor parent. The CO39 variety was used as a susceptible control for rice blast evaluation.

### Molecular markers and PCR amplification

Adoption of MAS was facilitated by using simple sequence repeat (SSR) marker RM527 for *Pi2* (Chen et al. 2008). Total DNA was extracted according to the procedure of Dellaporta et al. (1983). PCR reactions were performed on a MyCycler™ thermal cycler (BIO-RAD USA). Each 20 μl PCR reaction mixture contained 20 ng genomic DNA, 10 mM Tris–HCl (pH 9.0), 50 mM KCl, 2.5 mM MgCl_2_, 2 mM dNTPs, 10 μM each of the primer pair and 1 unit Taq DNA polymerase. Template DNA was initially denatured at 94°C for 5 min prior to 35 cycles of denaturation at 94°C (30s), annealing at 55°C (30s), and extension at 72°C (45 s). At the final step, the reaction mixture was incubated at 72°C for 5 min before the completion. The amplified products were then electrophoretically resolved on a 4% polyacrylamide gel in 1 × TBE buffer.

### Rice blast evaluation

The newly developed TGMS lines, C815S (TGMS gene donor), VE6219 (blast resistance gene donor) and CO39 (blast susceptible CK) were inoculated in greenhouse by 62 isolates of *M. oryzae* from Zhejiang and Guangdong provinces, China. Leaf and neck blast resistance were identified under natural conditions by planting in the rice blast disease hotspot location, the Wangjia village of Yuan-An county, Hubei, China, where rice blast diseases were epidemic every year. The disease resistance reaction both in greenhouse and under natural fields were carried out according to standard evaluation system for rice (SES) (IRRI 2002).

### Genetic background profiling by RICE6K

RICE6K, a whole-genome single nucleotide polymorphism (SNP) array, was used for genetic background profiling of newly developed TGMS line (Yu et al. 2014). RICE6K was developed based on Infinium technology, using representative SNPs selected from more than four million SNPs identified from resequencing data of more than 500 rice landraces. It contains 5102 SNP and InDel markers, about 4500 of which were of high quality in the tested rice lines producing highly repeatable results. For each line, total DNA was extracted from 20 dry seeds. DNA amplification, fragmentation, chip hybridization, single base extension, staining and scanning were conducted by Life Science and Technology Center, China National Seed Group Co., LTD (Wuhan, China), according to Infinium HD Assay Ultra Protocol (http://www.illumina.com/).

### Characterization of TGMS lines for fertility-sterility alteration under growth chamber

100 seeds per line were sown at the nursery plots of Experimental Farm of HAU on May 10 of 2012. Uniform and healthy rice seedlings at the 5-leaf stage (about 25 days after sowing) were selected for transplanting of five single plants per hill per plastic pot and each plant was labeled with plastic tags. Care was taken with proper crop management to allow the plants in each plot to grow well and be uniform. Five growth chambers (Model: ZSX1500GS manufactured by The Shanghai Jing Wins and Scientific Equipment Co., Ltd., China) were adjusted for dry runs, one week prior to their actual use for the experiments. Light duration and relative humidity level was set to 14 h and 75% uniformly to all the five growth chambers while the daily mean temperatures were set for 21°C, 22°C, 23°C, 24°C and 25°C, respectively. The temperature and light duration were carefully programmed to follow diurnal patterns at the same time achieve the required daily mean temperature and day light conditions. The plants were placed in growth chambers from 5 to 16 days after panicle initiation primordial stage. The plants were moved out from the growth chambers after 12 days of temperature treatment. The pollen grains collected from top five spikelets from each panicle per plant that headed during 5–16 days after the end of controlled growth chamber environment treatment were observed under microscope. The pollen sterility of each panicle was recorded according to the classification of pollen morphology upon IK-I staining of pollen grains (Virmani et al. 2003). The line with more than 99.5% of pollen sterility on an average was considered as completely sterile.

### Dynamic observation of pollen fertility-sterility alteration in the field

120 seeds in each line were sown at 15-day intervals at the Experimental Farm of HAU. Forty uniform and healthy rice seedlings at the 5-leaf stage were transplanted in fields with a spacing of 20 by 25 cm between plants and rows. The pollen fertilities in each line from the top five spikelets of primary panicles were investigated dynamically with two-day intervals under microscope from 30 June to 1 October. The pollen sterility data were recorded with average of five panicles from each line. The daily mean temperature data was provided by the Agricultural Meteorology Department of HAU. Analysis of pollen sterility data in relation to temperature weather charts was carried out to determine the CTP of fertility-sterility alteration under natural conditions.

### Evaluation of agronomic and rice grain quality traits

Thirty plants each of the newly developed TGMS lines and its recipient parent C815S were transplanted in the field during the winter season of 2011 at Rice Breeding Station of HAU, Lingshui County of Hainan province, China. Each plot comprised of three rows with 10 plants per row at planting density of 17 cm between plants and 20 cm between rows. The agronomic and rice quality traits were measured according to standard evaluation system for rice (IRRI, 2002). Five individuals in the middle of the second row in each plot were taken for measurements of agronomic traits, including days from sowing to heading, plant height, panicles per plant, panicle length, spikelets per panicle, spikelet fertility and weight of 1,000 grains. *T*-test was to detect statistical differences between developed TGMS line and recurrent parent. Harvested bulk seeds from each plot were used for analyzing rice grain quality traits that included milling rice percentage, rice grain length, grain length-width ratio, gel consistency and amylose content were also measured.

## Abbreviations

TGMS: Thermo-sensitive genic male sterile
CMS: Cytoplasmic male sterility
MAS: Marker assisted selection
SNP: Single nucleotide polymorphism
SSR: Simple sequence repeat
Indel: Insertion-deletion
CTP: Critical temperature point
DMT: Daily mean temperature
